# Antimicrobial Photodynamic Therapy: Self-Disinfecting Surfaces for Controlling Microbial Infections

**DOI:** 10.3390/microorganisms12081573

**Published:** 2024-08-01

**Authors:** Edith Dube

**Affiliations:** Department of Biological & Environmental Sciences, Walter Sisulu University, P/B X1, Mthatha 5117, South Africa; edube@wsu.ac.za; Tel.: +27-47-502-1952; Fax: +27-47-5022725

**Keywords:** self-disinfecting, pathogen, microbial, photosensitizers, public health

## Abstract

Microbial infections caused by bacteria, viruses, and fungi pose significant global health threats in diverse environments. While conventional disinfection methods are effective, their reliance on frequent chemical applications raises concerns about resistance and environmental impact. Photodynamic self-disinfecting surfaces have emerged as a promising alternative. These surfaces incorporate photosensitizers that, when exposed to light, produce reactive oxygen species to target and eliminate microbial pathogens. This review explores the concept and mechanism of photodynamic self-disinfecting surfaces, highlighting the variety and characteristics of photosensitizers integrated into surfaces and the range of light sources used across different applications. It also highlights the effectiveness of these surfaces against a broad spectrum of pathogens, including bacteria, viruses, and fungi, while also discussing their potential for providing continuous antimicrobial protection without frequent reapplication. Additionally, the review addresses both the advantages and limitations associated with photodynamic self-disinfecting surfaces and concludes with future perspectives on advancing this technology to meet ongoing challenges in infection control.

## 1. Introduction

Microbial infections, caused by bacteria, viruses, fungi, and parasites, pose a significant global threat to public health, manifesting in a spectrum of illnesses from mild to life-threatening conditions [1,2]. Certain environments, such as healthcare settings, schools, workplaces, public transportation systems, food and water sources, animal and agricultural environments, travel and tourism destinations, and disaster zones, are particularly vulnerable to infections. Factors contributing to this vulnerability include high population density, inadequate sanitation, close interpersonal contact, and compromised healthcare infrastructure [3]. According to Hui et al. (2024), in February 2024, 74 infectious diseases had a widespread impact across 236 countries globally. COVID-19 was notably pervasive, affecting every country, alongside other prevalent diseases such as smallpox, dengue fever, measles, and cholera. Among these, diseases with the highest fatality rates were Nipah virus, avian influenza A(H5N1), smallpox, Lassa fever, and Crimean–Congo hemorrhagic fever, contributing significantly to global mortality rates [4]. This shows the magnitude and varying severity of infectious diseases.

Disinfection is highly utilized in high-touch surfaces, public spaces, food preparation areas, agricultural facilities, and vulnerable environments as it reduces infections by eliminating harmful microorganisms on surfaces, equipment, and environments [5]. Conventional disinfection methods include chemical disinfectants like bleach (sodium hypochlorite) for surfaces in healthcare and homes; alcohol (ethanol or isopropanol) for hand sanitizers and small surfaces; hydrogen peroxide for healthcare equipment; quaternary ammonium compounds for hospitals, schools, and food areas; and phenolic compounds for healthcare and laboratories. Physical methods involve heat (autoclaving and boiling), UV radiation (disinfecting surfaces, air, and water), and dry heat (sterilizing instruments). Mechanical methods include filtration (air and liquid purification) and scrubbing (removing dirt before disinfection). Gaseous methods use ozone (disinfecting water, air, and surfaces) and ethylene oxide (sterilizing heat-sensitive equipment). These conventional methods are crucial for infection control [6,7]. 

However, although widely employed, these conventional disinfecting methods face significant limitations. They often require frequent disinfectant application to maintain efficacy, which is labor-intensive and time-consuming. Moreover, they commonly rely on harsh chemicals such as chlorine and quaternary ammonium compounds, posing health risks to humans and contributing to environmental pollution. According to Täubela et al., inefficient attempts to completely eradicate microbes from indoor surfaces lead to excessive use of disinfectants, raising concerns about promoting antimicrobial resistance and increasing indoor chemical loads [8]. Furthermore, the indiscriminate nature of these disinfectants can disrupt beneficial microbial ecosystems while promoting the development of resistant strains among pathogens. Their short-lived effectiveness and susceptibility to organic matter further diminish their reliability in maintaining sterile environments, especially in high-touch areas like hospitals and public facilities [7]. Therefore, adopting sustainable practices and enhancing infection control and sanitation measures is essential for effective disease management and safeguarding public health.

Innovative solutions like self-disinfecting surfaces offer promise in disease management and health improvement [9,10]. These surfaces are designed with antimicrobial properties to continuously eradicate or inhibit pathogen growth upon contact, thereby reducing microbial presence and preventing disease transmission [9,11]. By incorporating these self-disinfecting surfaces into infrastructure, such as floors, surfaces, tanks, ponds, nets, clothing, and equipment, the risk of disease spread can be minimized, promoting overall health and well-being. 

Antimicrobial photodynamic therapy (aPDT) is a promising technology that offers a novel approach to self-disinfecting surfaces, effectively and continuously deactivating pathogens without inducing resistance [12]. In aPDT, light-sensitive compounds, known as photosensitizers (PS), are irradiated with light of specific wavelengths, in an oxygenated environment, to generate reactive oxygen species (ROS) such as singlet oxygen and free radicals, which can destroy or inhibit the growth of pathogens [13,14]. In self-disinfecting surfaces, aPDT photosensitizers can be embedded or coated on the surface material, such that on exposure to light, they are activated, producing ROS, and disinfecting the surface [15,16,17]. This approach offers a promising means of continuously disinfecting surfaces [17], thereby reducing the risk of microbial contamination and infection. For instance, Eichner et al. conducted an extensive study to evaluate the effectiveness of the photodynamic coating system DYPHOX^®^, which contains photosensitizers, in reducing bacterial contamination in a hospital setting. The DYPHOX^®^ system was applied to a variety of hospital surfaces, aiming to minimize bacterial burden and improve overall hygiene. The study’s findings revealed a significant reduction in bacterial contamination on the treated surfaces. Furthermore, the antimicrobial properties of the DYPHOX^®^ coating were found to be stable and effective for at least six months, underscoring its potential for long-term application in healthcare environments to enhance hygiene and reduce the risk of infections [18]. 

Additionally, the efficacy of these coatings was not limited to hospital settings. Another study demonstrated that the DYPHOX^®^ coatings significantly decreased the mean bacterial burden on various inanimate surfaces in urban buses under real-life conditions [19]. This suggests that photodynamic self-disinfecting surfaces could be a valuable tool not only in healthcare settings but also in public transportation and other high-contact areas to help control the spread of harmful bacteria and maintain cleaner environments.

This review explores photodynamic self-disinfecting surfaces as a cutting-edge approach to combat microbial infections caused by bacteria, viruses, fungi, and parasites across diverse environments. It begins by introducing the concept and mechanism of photodynamic self-disinfecting surfaces, focusing on antimicrobial photodynamic therapy. This section explains how photosensitizers integrated into surfaces generate reactive oxygen species upon exposure to light, effectively targeting and eliminating microbial pathogens. The types and ideal characteristics of PS used in aPDT self-disinfecting surfaces to combat microorganisms are explored together with the light sources utilized. The review also discusses the advantages and limitations associated with photodynamic self-disinfecting surfaces.

The materials for this study were sourced from published articles on Scopus, Google Scholar, and ResearchGate, using keywords such as self-disinfecting, public health, infections, microbial, photosensitizers, and light among others. Articles were initially screened by reviewing abstracts, followed by a thorough reading of full papers to identify relevant studies. Key information was extracted from these selected articles and incorporated into this review.

## 2. Antimicrobial Photodynamic Therapy

**Figure 1:**
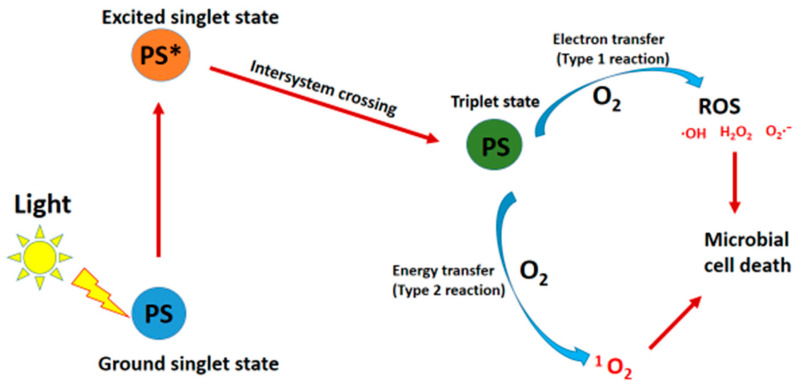
An illustration of type 1 and type 2 aPDT reactions.

aPDT is a technique utilized to eradicate pathogenic microorganisms by harnessing the combined effect of PS and light, in an oxygen-rich environment [12]. In areas affected by bacterial infections, the PS is applied, followed by exposure to light of specific wavelengths. This exposure triggers the transition of the PS from its ground state to an excited state (PS*), as depicted in Figure 1. This excited state is transient and can undergo intersystem crossing (ISC) to a triplet state. In this state, the PS can engage in either type 1 or type 2 reactions.

In type 1 reactions, the triplet state PS transfers electrons or protons to surrounding molecules within the microorganism cells, generating free radicals that react with molecular oxygen (O_2_) to form ROS like hydrogen peroxide (H_2_O_2_), superoxide (O_2_^–^), and hydroxyl (•OH) radicals. These ROS then interact with bacterial biomolecules, causing damage and eventual cell death. Conversely, in type 2 reactions, the triplet state PS transfers energy to ground-state molecular oxygen, yielding singlet oxygen (^1^O_2_) that induces cellular damage and death by interacting with biomolecules within the cell [20,21]. 

On self-disinfecting surfaces, PSs can also inactivate microbes without directly penetrating or contacting the cells. Instead, ^1^O_2_ generated in sufficient quantities near the microorganism’s outer membrane diffuses into the cell, destroying its components. This indirect mechanism enables effective antimicrobial action even without direct contact between the PSs and the bacterial cells, making self-disinfecting surfaces highly efficient at eliminating pathogens [22,23]. 

Detecting ROS on self-disinfecting surfaces is essential for evaluating antimicrobial activity. Various methods are employed based on the type of ROS present. Fluorescent probes detect ROS by measuring fluorescence changes. Each fluorescent probe is designed to detect specific types of ROS [24,25]. For instance, Singlet Oxygen Sensor Green detects ^1^O_2_, producing a fluorescent signal upon interaction. 4-Hydroxyphenylfluorescein is used for •OH, yielding a fluorescent product. Amplex Red detects H_2_O_2_, forming the fluorescent compound resorufin in the presence of horseradish peroxidase. Nitroblue tetrazolium detects O_2_^−^, producing colored or fluorescent products upon reduction. These probes allow for precise and sensitive ROS detection, aiding in the study of the efficacy of antimicrobial treatments [25,26,27]. Additionally, singlet oxygen can be detected through photoluminescence, which measures luminescent emission from decaying singlet oxygen [28]. Colorimetric assays are also utilized to measure absorbance changes resulting from ROS-induced color changes. Oxidation-reduction indicators, such as 3,3′-Diaminobenzidine, form visible precipitates upon reacting with ROS. Additionally, electrochemical sensors can detect ROS by measuring electrical signals generated on ROS-sensitive electrodes [24]. Each method offers specific advantages in sensitivity and accuracy, contributing to a comprehensive assessment of antimicrobial properties on surfaces. Table 1 shows the photosensitizers used in aPDT self-disinfecting surfaces to combat microorganisms, including bacteria, viruses, and fungi.

The information in Table 1 highlights the diverse range of PSs and light sources used in aPDT to target and inhibit various microorganisms (bacteria, viruses, and fungi), demonstrating the potential of self-disinfecting surfaces in mitigating microbial contamination and infection across different environments. This versatility allows for tailored approaches in different settings, enhancing the effectiveness of infection control measures. By employing specific PSs and light sources, it is possible to achieve effective microbial control for various pathogens, thus reducing the risk of infections and promoting healthier environments in healthcare, public transportation, and other high-contact areas.

### 2.1. Photosensitizers

PSs play an important role in aPDT reactions as absorbers of light energy [20]. Various PSs, such as phthalocyanines, porphyrins, boron dipyrromethenes (BODIPYs), 9-aminoacridine, thionine, azure A, graphitic carbon nitride, methylene blue, toluidine blue O, and Rose Bengal, have shown efficacy against microbes upon light activation when embedded on different surfaces [12,15,17,28,29,30,31,32,33,34,35,36,37,38,39,40,41,42,43,44,45,46]. Like other disinfecting materials, PSs in self-disinfecting surfaces should possess certain ideal properties. 

**Figure 2:**
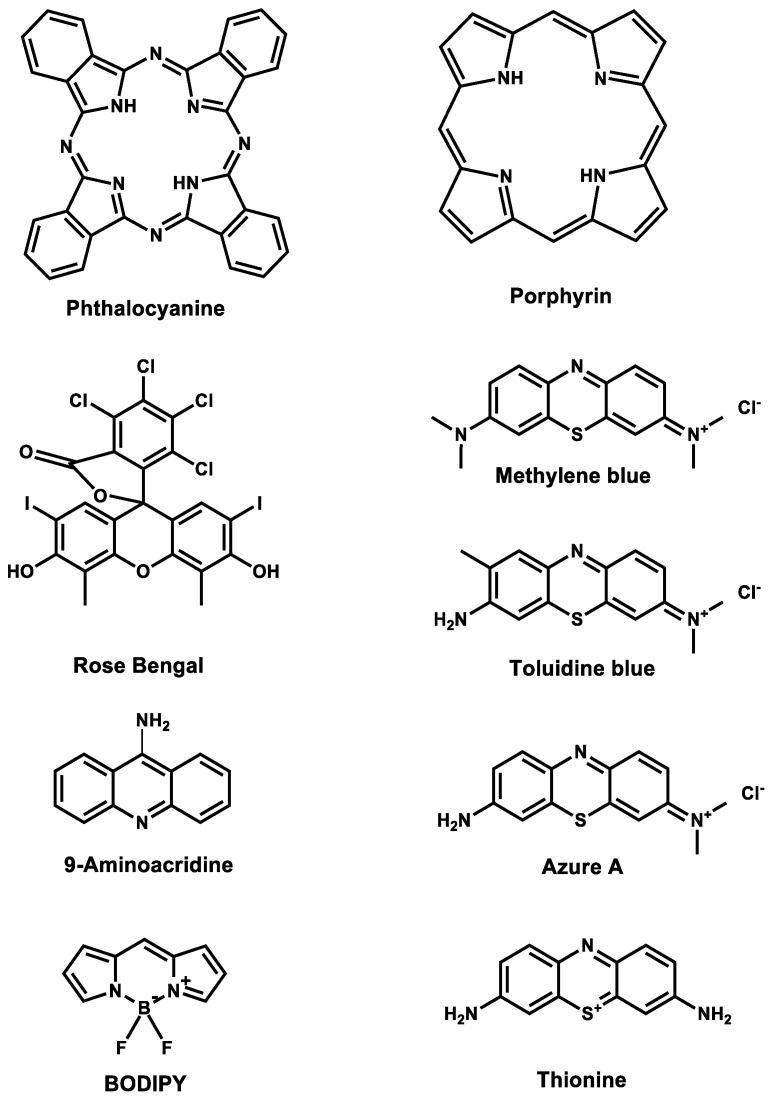
PSs employed in self-disinfecting aPDT surfaces.

An effective PS should efficiently generate ROS upon light activation to ensure effective microbial inactivation. To achieve this, the PS must be designed with structural features such as conjugated double bonds, aromatic rings, and electron-donating or withdrawing substituents, which can enhance light absorption and facilitate efficient energy transfer to molecular oxygen, leading to ROS generation [47,48,49]. Figure 2 depicts the structural characteristics of some PSs employed in self-disinfecting aPDT surfaces. 

Each of these photosensitizers, composed of conjugated double bonds, aromatic rings, and electron-donating or withdrawing substituents, exhibits distinctive structural characteristics that contribute to their photodynamic properties and antimicrobial applications. Phthalocyanines and porphyrins are large, planar molecules with metal ions at their centers, enabling strong light absorption and ROS generation [12]. BODIPYs have a rigid, planar boron-dipyrromethene core, making them highly efficient at producing singlet oxygen upon light exposure [50]. 9-Aminoacridine consists of three fused aromatic rings, forming the acridine core. This extended π-conjugated system is crucial for its photophysical properties, allowing it to absorb light efficiently. It intercalates with DNA, targeting nucleic acids [44]. Thionine and Azure A are phenothiazine derivatives, generating ROS upon light activation [38,45]. Graphitic carbon nitride is a two-dimensional layered semiconductor that generates ROS under visible light. Methylene blue and toluidine blue O are thiazine dyes, effective in redox cycling to produce ROS. Rose Bengal, a xanthene dye with substituted phenols, efficiently generates singlet oxygen upon light exposure [12]. The diverse structures and electronic properties of PSs influence their ROS generation efficiency, enabling tailored applications in self-disinfecting aPDT surfaces. Additionally, the chemical composition of PSs, including functional groups, is vital for binding to surfaces or polymers. While charged groups can enhance interactions with microbial membranes, specific functional groups improve compatibility with polymer matrices. The balance between hydrophobic and hydrophilic properties is crucial, as hydrophobic PSs interact effectively with lipid-rich microbial membranes, and hydrophilic PSs enhance solubility and distribution in aqueous environments [51,52]. This solubility allows them to be evenly dispersed across surfaces, ensuring comprehensive coverage and more effective microbial inactivation. The attachment method, whether through covalent bonding or physical adsorption, also impacts the stability and longevity of the coatings, with covalent attachment generally providing more durable and stable results [53,54,55]. For instance, a hydroxy-functionalized tetraphenylporphyrin was covalently linked to an acrylate and metalated with zinc, creating a zinc tetraphenylporphyrin acrylate monomer. This monomer achieved high incorporation rates (up to 95%) in various well-defined linear copolymer formulations. The attachment of the PS to the polymer chains did not hinder singlet oxygen generation, allowing the previously insoluble tetraphenylporphyrin to disperse well in aqueous environments. This structural modification significantly enhanced the antimicrobial properties of the polymers, providing substantial activity against Gram-positive *S. aureus*. It imparted both bacteriostatic and bactericidal properties to previously inactive *N*-hydroxyethyl acrylamide polymers and increased the efficacy of previously active acrylamidopropyl trimethylammonium chloride polymers. In particular, polymers previously inactive against *S. aureus* gained significant bactericidal activity with the incorporation of the photosensitizer monomer [52]. These findings demonstrate the crucial role of photosensitizer structure in determining the antimicrobial capabilities of surface coatings. Optimizing these structural elements (chemical composition, hydrophobicity/hydrophilicity, and attachment mechanisms) is essential for enhancing the antimicrobial properties and durability of PS when used in coatings or polymers [54].

Moreover, an ideal PS should exhibit potent antimicrobial properties against a broad spectrum of pathogens commonly found in different environmental settings, including bacteria, viruses, fungi, and protozoa. Various PSs have demonstrated aPDT activity against a range of microbes. For instance, Li et al. reported the aPDT activity of meso-pyridinium 1,3,5,7-tetramethyl BODIPY against *Staphylococcus aureus*, *Escherichia coli*, *Candida albicans*, and methicillin-resistant *S. aureus* when exposed to a light dose of 81 J/cm^2^ [56]. Another BODIPY-based photosensitizer, 2,6-diiodo-1,3,5,7-tetramethyl-8-(N-methyl-4-pyridyl)-4,4′-difluoroboradiazaindacene (DIMPy-BODIPY), was found to possess antiviral, antibacterial, and antifungal photodynamic activity at nanomolar concentrations and short illumination times (fluence rate of 65 ± 5 mW/cm^2^) [57]). Rose Bengal, as shown in Table 1, has demonstrated activity against bacteria [29,30,31,32], viruses [35], and fungi [31,41] in self-disinfecting aPDT surfaces. This shows the potential of diverse PSs in combatting a wide range of pathogens, emphasizing their role in the development of self-disinfecting surfaces for enhanced microbial control. 

Furthermore, PSs are prone to irreversible damage or degradation upon light exposure, known as photobleaching, which can diminish their ability to generate ROS and reduce their effectiveness in aPDT applications [58,59]. Therefore, an ideal PS should remain stable and maintain its antimicrobial activity upon light exposure, enabling effective and prolonged treatment without degradation [60,61]. For instance, Rodrigues et al. demonstrated the stability of PSs, such as bacteriochlorin metoxi and bacteriochlorin trizma, both derivatives of bacteriochlorophyll. These PSs remained unchanged under experimental conditions (45 J/cm^2^) and did not exhibit a decrease in their absorbance levels [60]. Ensuring the stability and effectiveness of PSs is crucial throughout the aPDT treatment. Strategies to reduce photobleaching include optimizing the chemical structure of PSs or employing protective formulations. Maliszewska et al. utilized biogenic gold nanoparticles to decrease the photobleaching of methylene blue, enhancing its aPDT efficacy [62]. PSs like zinc-tetra(4-*N*-methylpyridyl)porphine, methylene blue, and Rose Bengal have demonstrated activity against the Human coronavirus strain HCoV-229E even after prolonged light exposure for 4 weeks [35]. Similarly, phenoxy-substituted zinc phthalocyanines maintained activity against *Bacteriophage Qβ* for over 6 months under continuous exposure to room light [17], highlighting the importance of stable PSs in maintaining efficacy over extended periods, especially in self-disinfecting aPDT surfaces. 

The longevity of PSs on surfaces is essential for sustained antimicrobial effectiveness [63,64]. In addition to resisting photodegradation, PSs must also resist degradation from other environmental factors such as temperature changes, pH variations, oxygen, and moisture. They also need to withstand cleaning agents and mechanical wear, particularly on high-touch surfaces. To prevent PS degradation, several strategies have been employed, including encapsulating them in protective layers, controlling pH and temperature, using stabilizing agents and nanoparticles and employing effective binding mechanisms. For example, curcumin, a commonly used PS, can degrade in alkaline conditions, producing ferulic acid and feruloyl methane [65]. However, encapsulating curcumin has been shown to reduce its degradation and enhance its effectiveness in aPDT [66]. Manathanath et al. also demonstrated the effectiveness of incorporating PSs such as 5,15-di(3′-*N*-methylpyridyl)-10,20-bis(pentafluorophenyl) porphyrin into gelatin/polyurethane electrospun membranes for bacterial eradication through photoinactivation [67]. As shown in Table 1, various polymers or coatings have also been employed to shield PSs and promote the longevity of self-disinfecting aPDT surfaces. Ghareeb et al. conducted a comprehensive study on the long-term viability of a spray coating made from the polymer *N*-methyl-4(4′-formyl-styryl)pyridinium methosulfate acetal poly(vinyl alcohol) (SbQ-PVA), combined with either zinc-tetra(4-*N*-methylpyridyl)porphine, methylene blue, or Rose Bengal. Remarkably, even after 4 weeks, all three coatings effectively inactivated HCoV-229E down to the detection limit and maintained their antimicrobial activity through multiple wash and assay cycles [35]. This demonstrates their robustness and long-lasting effectiveness. Covalent bonding with polymers and coatings provides more durable PS attachment than non-covalent interactions, further extending their lifespan. Protective coatings and uniform application can further enhance the longevity of PSs. Optimizing these factors ensures reliable and long-lasting antimicrobial protection [68].

The PS should also demonstrate non-toxicity at effective concentrations, typically indicated by the absence of aPDT effects under dark conditions [69,70]. For instance, Zada et al. did not observe any reduction in *Pseudomonas aeruginosa* colonies following treatment with methylene blue alone, without irradiation, indicating the lack of antimicrobial activity of the PS when used alone [69]. This aspect is crucial as the PS must not endanger other organisms, including humans and animals.

The solubility of PSs is crucial when embedding them in polymers or coatings, as it significantly affects the distribution, stability, and overall effectiveness of the antimicrobial properties of the final material. Proper solubility ensures that the PS is evenly distributed throughout the polymer matrix or coating solution, which is crucial for consistent antimicrobial activity across the entire surface. Additionally, soluble PSs are less likely to precipitate or aggregate, which helps maintain a stable and active form, thereby prolonging the material’s antimicrobial effectiveness [71]. Furthermore, the solubility of the PS can influence the adhesion of the coating to various surfaces, with a well-dissolved PS enhancing bonding and adherence, ensuring the coating’s durability and effectiveness. For instance, the water-soluble photosensitizer ({4,4′,4″,4‴-(29*H*,31*H*-phthalocyanine-1,8,15,22-tetrayl-κ4N29, N30, N31, N32) tetrakis [1-methylpyridiniumato(2-)]} zinc(4+) tetraiodide), exhibited exceptional adhesion to the filter paper as a photoactive antimicrobial surface. This ensured that the PS remained securely attached to the paper without migrating or leaching into the surrounding solution [72]. This property is crucial for maintaining the integrity and effectiveness of the PS especially under environmental conditions such as temperature, humidity, and exposure to cleaning agents. PS that is too soluble may leach out or degrade quickly, reducing the effectiveness [39,73]. Soluble PSs can be processed and applied more easily through various methods, including spraying, dipping, spin-coating, mixing with polymers, embedding in matrices, or applying as a film. For instance, Rose Bengal, toluidine blue O, and methylene blue have been immobilized into the hydrophobic polymer poly (vinylidene fluoride) on a polyethylene sheet to inactivate *S. aureus* and *E. coli*. Goniometry revealed all surfaces were hydrophobic, while photodynamic analysis indicated significant ROS formation after 30 min of illumination at 1.46 mW cm^–2^ [74]. Hydrophobic surfaces can provide stability and durability across various conditions.

Optimizing the properties of PSs is essential for their effective application in self-disinfecting aPDT surfaces, ensuring strong antimicrobial activity, stability under various environmental conditions, and safety towards non-target organisms. Further research and development in PS modification and formulation strategies will continue to advance the efficacy and safety of aPDT as a promising approach for microbial control.

### 2.2. Light

Light plays a crucial role in aPDT by activating PS to produce ROS and induce microbial inactivation [13]. The choice of light source for aPDT depends on factors such as the PS’s absorption spectrum, treatment area, and depth of penetration required. For instance, Pérez-Laguna et al. utilized an LED lamp emitting red light (625 nm) and a white metal halide (WMH) lamp emitting broad-spectrum white light (420–700 nm) at a fluence of 18 J/cm^2^, in conjunction with methylene blue as a PS for aPDT against *Candida albicans*, *Candida parapsilosis*, and *Candida krusei*. The WMH lamp achieved photoinactivation of *Candida* spp. in 3 min and 25 s, compared to 43 min for the LED lamp, attributed to the former’s higher irradiance (90 mW/cm^2^) relative to the latter (7 mW/cm^2^) [75], highlighting the importance of light source selection.

The light sources utilized in the studies shown in Table 1, cover a wide range of wavelengths and intensities tailored to the specific PS and microbial target. Various light sources such as white LED light, laser light, Xenon lamps, LED lamps emitting specific wavelengths, and artificial sun irradiation were employed. These sources are selected based on their ability to match the absorption spectra of the PS used, thereby optimizing ROS generation for effective aPDT.

#### 2.2.1. Light-Emitting Diodes

LEDs are widely used in aPDT due to their versatility, energy efficiency, cost-effectiveness relative to other light sources, and controllability. They offer tunable wavelengths that can align with the absorption spectrum of different PSs, enabling precise activation [13,76]. For instance, in aPDT studies targeting *Staphylococcus aureus* and *Pseudomonas aeruginosa*, Farzamian et al. utilized a red LED light source emitting at 660 nm (with a power density of 20 mW/cm^2^) in order to align it with the maximum absorption of the PS methylene blue [77]. In a related study, an LED light emitting at 515 ± 10 nm (200 J/cm) was utilized in order to align it with the maximum absorption of the PS Rose Bengal [31]. This shows the capacity of LEDs to be tailored to match the absorption characteristics of a PS.

#### 2.2.2. Lasers

Laser sources emit precise and intense light beams, allowing for precise targeting of specific areas or pathogens [76]. For instance, Chakraborty et al. fabricated a low-cost, handheld, LED-based device as a light source for aPDT against intranasal infections. When combined with a PS containing methylene blue and potassium iodide at 655 nm, this device demonstrated in vitro photodynamic inactivation of pathogens commonly found in the nasal cavity, including, methicillin-resistant *Staphylococcus aureus*, antibiotic-resistant *Klebsiella pneumoniae*, multi-antibiotic-resistant *Pseudomonas aeruginosa*, *Candida* spp., and SARS-CoV-2 [78].

Lasers emit light with narrowband wavelengths, which is advantageous for activating PS with narrow absorption bands [76,79]. Additionally, lasers can emit light at tailored wavelengths to align with the absorption spectrum of the PS used in aPDT, ensuring optimal activation and maximizing ROS production for enhanced antimicrobial efficacy [76]. For instance, in studies involving the PS Rose Bengal, which has an absorption maximum of 559.1 nm, aPDT investigations were conducted using a 535 nm wavelength laser to ensure optimal aPDT effect [80,81]. Furthermore, laser sources generate high-intensity light beams that effectively activate PS, leading to microbial inactivation. This focused energy delivery enhances ROS generation, resulting in potent antimicrobial effects [13,79,82]. However, the use of lasers as a light source for aPDT may present limitations such as high costs, limited penetration depth, and safety concerns (as lasers can pose risks to operators and non-target aquatic organisms).

#### 2.2.3. Broadband Lamps

Broadband lamps emit light across a wide spectrum of wavelengths, including visible and near-infrared regions [76,83]. However, adverse effects such as heat generation by infrared light and tissue damage by ultraviolet light can cause problems. For this reason, spectral filters are usually applied to cut off wavelengths not matching the absorption of the PS [13]. For instance, Solarte et al. in their aPDT studies utilized a combination of visible light and water-filtered infrared A (with wavelength range from 570 nm to 1400 nm) as the light source. The applied irradiance was approximately 48 mW/cm^2^ in the visible range and 152 mW/cm^2^ in the water-filtered infrared-A range, resulting in a total irradiance of 200 mW/cm^2^. This irradiance was applied to bacterial strains for 5 min in the presence of Indocyanine Green, which exhibits maximal light absorption at 800 nm as the PS. This approach resulted in significant antimicrobial activity against bacterial pathogens [84]. Broadband lamps offer versatility and cost-effectiveness, making them well-suited for large-area treatments and scenarios where precise wavelength control is not as crucial. These lamps are especially advantageous for covering extensive surfaces efficiently, providing uniform illumination over a wide area [13,76,79]. As a result, they are suitable for aPDT disinfection in various environmental settings.

#### 2.2.4. Sunlight

Natural sunlight has the potential to serve as a sustainable and environmentally friendly light source for aPDT, particularly in outdoor or field applications [13,85]. Pérez-Laguna et al. demonstrated the effectiveness, safety, and efficiency of daylight aPDT using methyl blue as a PS in treating dermatophytosis caused by *Arthroderma vanbreuseghemii* in ewes [86]. Sunlight offers a readily available and cost-effective alternative to artificial light sources, potentially reducing operational costs. Additionally, sunlight can illuminate large areas with high uniformity and encompass a broad spectrum of wavelengths, enhancing the effectiveness of aPDT treatments by targeting various microbial pathogens [13]. Wang et al. developed a sunlight-driven aPDT method using a cationic conjugated microporous polymers-based coating, which showed promising results [87]. However, it is important to note that sunlight’s intensity and spectral composition can vary based on factors like time of day, weather conditions, and location, necessitating careful consideration for consistent and effective daylight aPDT treatment.

### 2.3. Oxygen

Oxygen is one of the major components that play a crucial role in aPDT as it is involved in the generation of ROS, which is responsible for the antimicrobial effect, as illustrated in Figure 1. Therefore, the presence of sufficient oxygen levels is critical for the efficacy of aPDT. Though oxygen occurs naturally in the environment and cells, oxygenation strategies, such as aeration, and oxygen supplementation are often utilized to ensure adequate oxygen levels for aPDT [88]. For instance, Niu et al. fabricated a Chlorin e6 and perfluorodecalin nanoemulsion, with perfluorodecalin utilized due to its high affinity to oxygen, to increase oxygen levels in cells, ultimately increasing the levels of ^1^O_2_ during aPDT [89].

## 3. Impact of Microbial Structures on Antimicrobial Photodynamic Therapy Effectiveness

Microbial structures are diverse, and their responses to aPDT agents can vary based on these structural differences [90]. Understanding the diverse structures of microbes and how they interact with photodynamic agents is crucial for designing effective aPDT strategies. The ability of PS to penetrate microbial structures, bind to specific targets, and generate ROS upon light activation determines the overall antimicrobial efficacy of aPDT [12].

### 3.1. Bacteria

Bacteria are classified into Gram-positive or Gram-negative categories. Gram-negative bacteria, such as *P. aeruginosa*, *E. coli*, and *A. baumannii*, and Gram-positive bacteria, including *S. aureus*, *S. epidermidis*, *S. pneumoniae*, and *E. faecalis*, have been effectively inactivated by PSs on self-disinfecting surfaces [28,29,30,31,32,33,34,35,36,37,38,39,40,41,42,43,44,45]. Gram-positive bacteria have a thick peptidoglycan layer in their cell walls, which retains PSs more effectively due to its thickness and structure. In aPDT, PSs penetrate this cell wall and accumulate within the bacterial cell. Upon light activation, ROS are produced, damaging cellular components such as the cell wall, membrane, proteins, and DNA. In contrast, Gram-negative bacteria have a thin peptidoglycan layer surrounded by an outer membrane containing lipopolysaccharides (LPS), which can act as a barrier to PSs [90]. This outer membrane can impede the uptake of PSs. However, using PSs that can bind to LPS or employing strategies to disrupt the outer membrane can enhance their effectiveness. Since LPS molecules are negatively charged due to phosphate groups, PSs with positive charges can electrostatically interact and bind to the negatively charged LPS.

For instance, a novel polymyxin B (PMB)-targeted liposomal hematoporphyrin monomethyl ether (HMME) photosensitizer (HMME@Lipo-PMB) has been developed to enhance antimicrobial photodynamic therapy. This innovative photosensitizer specifically targets and binds to the lipopolysaccharides (LPS) in Gram-negative bacteria, leveraging both the photodynamic therapy effect of HMME and the antimicrobial properties of PMB. PMB, a positively charged lipopeptide antibiotic, binds to the negatively charged phosphate groups in the Gram-negative bacterial membrane. Encapsulating HMME within a liposomal structure conjugated with PMB ensures effective delivery and binding to bacterial cells, enhancing ROS generation upon light activation and improving overall treatment efficacy. This approach has shown a significant reduction in bacterial viability in vitro and completely eradicated burn infections induced by MDR *A. baumannii* in vivo [91]. Additionally, Chaves et al. conjugated porphyrin macrocycles with triphenylphosphonium units, creating a series of effective cationic porphyrin-based PSs for aPDT. The presence of positive charges at both the porphyrin macrocycle and triphenylphosphonium moieties significantly enhances the photodynamic activity of these porphyrin-based PSs against even Gram-negative bacterial strains that are typically more resistant to aPDT [92]. The ROS generated during antimicrobial photodynamic therapy (aPDT) can damage the outer membrane, peptidoglycan layer, inner membrane, and intracellular components, leading to bacterial cell death.

Functionalizing PSs with specific ligands that have a high affinity for LPS, such as peptides or molecules known to bind LPS, can further enhance their targeting and effectiveness. For example, Yang et al. conjugated peptides with PSs and showed that this approach was effectively bactericidal against *E. coli* while keeping the peptide’s weakly helical shape intact [93].

Smaller PSs can more easily penetrate the bacterial outer membrane compared to larger molecules, providing improved access to LPS and enhancing antimicrobial efficacy. To further boost their effectiveness, PSs are often linked to nanoparticles to improve their uptake by bacterial cells. For example, mesoporous silica nanoparticles loaded with a squaraine dye as a photosensitizer demonstrated significantly greater antibacterial activity against both *S. aureus* and *E. coli* compared to the photosensitizer alone [94], highlighting the benefits of enhanced cellular uptake facilitated by the nanoparticles.

### 3.2. Viruses

The structure of viruses can be categorized into non-enveloped and enveloped types. Non-enveloped viruses have a protein coat called a capsid, made up of subunits known as capsomeres, protecting their DNA or RNA genome. This genome can be single-stranded or double-stranded. In contrast, enveloped viruses have an additional lipid bilayer, derived from the host cell membrane, which surrounds their capsid. This envelope contains embedded glycoproteins essential for viral entry into host cells [95,96,97].

In aPDT, PSs are designed to target specific viral structures. For non-enveloped viruses, PSs bind to the capsid proteins [98]. One such PS, 5,10,15,20-tetrakis (1-methyl-4-pyridinio) porphyrin tetra p-toluene sulfonate (TMPyP), has been shown to rapidly inactivate the bacteriophage MS2. When exposed to light at 32 mW/cm², TMPyP inactivates MS2 within one minute. This highlights TMPyP’s effectiveness in targeting and inactivating non-enveloped viruses by generating ROS upon light activation, causing oxidative damage to the viral capsid [99]. For enveloped viruses, they target the lipid envelope and its glycoproteins. Pourhajibagher et al. demonstrated that PSs curcumin, quercetin, and riboflavin can efficiently bind to the D8L protein in the Monkeypox virus, a crucial component of its structure, with strong binding affinity. The D8L protein, located in the viral envelope, is essential for the virus’s ability to infect host cells. Upon light activation, these PSs generate ROS that oxidize and damage both the viral envelope and capsid. This oxidative damage disrupts the viral structures, impairing the virus’s ability to infect host cells and leading to its inactivation [100]. Thus, aPDT effectively targets and inactivates both non-enveloped and enveloped viruses by disrupting their structural integrity.

### 3.3. Fungi

The fungal cell wall, composed of chitin, glucans, and mannoproteins, provides rigidity and protection. The cell membrane contains ergosterol instead of cholesterol, making it a target for many antifungal treatments. In aPDT, PSs penetrate the fungal cell wall and localize within the cell membrane or cytoplasm. Upon light activation, these PSs generate ROS that oxidize ergosterol, disrupt membrane integrity and damage intracellular components. This oxidative damage to the cell wall, membrane, and internal structures ultimately leads to fungal cell death [101,102]. The effectiveness of aPDT against fungi lies in the PS’s ability to penetrate the cell wall and localize within the cell, where the generated ROS induce significant damage, ensuring the death of the fungal cells. Research by Prandini et al. demonstrated that modifying zinc (II) phthalocyanine with the pyridinium groups enhances its properties. This derivatization results in increased singlet oxygen quantum yield, improved PS uptake, and more extensive distribution within the cytoplasm, all contributing to more effective fungal cell eradication [103].

## 4. Photodynamic Self-Disinfecting Surfaces

Photodynamic self-disinfecting surfaces are an innovative strategy for combating microbial contamination in different environments. They offer continuous antimicrobial protection [10,104]. The studies outlined in Table 1 showcase a diverse range of photodynamic disinfecting surfaces utilizing various PSs and light sources tailored for specific microbial targets. For instance, Rose Bengal, methylene blue, and various phthalocyanines are employed alongside light sources such as white LED lamps, lasers, Xenon lamps, and specific LED wavelengths, each selected to maximize ROS generation and microbial inactivation. Surfaces include polymer-coated materials like polyethylene terephthalate (PET), cotton fabrics, and ion exchange resins, among others. This diversity highlights the adaptability of PDT in creating self-disinfecting surfaces effective against a wide array of bacteria, viruses, and fungi [12,15,17,28,29,30,31,32,33,34,35,36,37,38,39,40,41,42,43,44,45,46].

There is a wide range of surfaces prone to microbial colonization and biofilm formation, including medical devices, hospital surfaces, food processing equipment, dental instruments, water systems, public facilities, industrial settings, household surfaces, and various environmental settings [105,106,107]. Lo et al. using polycarbonate plastics, demonstrated that plastisphere-biofilms in the environment may encourage the growth and spread of harmful pathogenic bacteria such as *Pseudoalteromonas* and *Pseudomonas* [108]*,* highlighting the importance of continuous surface disinfection.

**Figure 3:**
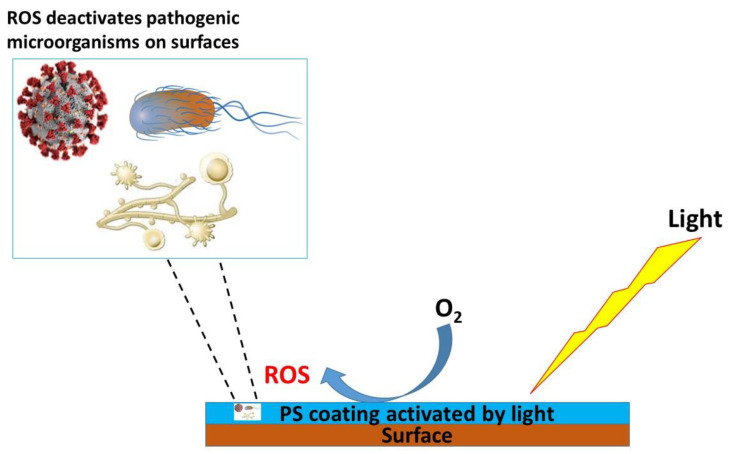
An illustration of aPDT reactions on PS-coated surfaces resulting in the inactivation of pathogenic microbes.

For photodynamic self-disinfection, the surface materials are engineered to possess inherent antimicrobial properties through the incorporation of the PSs [10,104]. These can either be sprayed, dipped, brushed, or roll-coated with a PS-containing coat, depending on the nature of the surface. Ghareeb et al. studied the antimicrobial efficacy of a spray coating made of the UV photocross-linked polymer *N*-methyl-4(4′-formyl-styryl) pyridinium methosulfate acetal poly (vinyl alcohol) (SbQ-PVA) embedded with a PSs, which is either methylene blue, Rose Bengal or zinc-tetra(4-*N*-methylpyridyl)porphine. The spray coating on irradiation with visible light (400–700 nm, 65 ± 5 mW/cm^2^) for 60 min, inactivated more than 90% of the microbes studied (methicillin-susceptible and human coronavirus strain). The inactivation was observed on exposure to ambient indoor room lighting even after 4 weeks [35], showing that the coatings remain effective for a longer period, on exposure to light. These surfaces can generate ROS, such as singlet oxygen, upon exposure to light, effectively destroying a broad spectrum of microorganisms, including bacteria, viruses, and fungi [104,109], as illustrated in Figure 3.

For instance, a phenoxy-substituted zinc phthalocyanine incorporated into a cellulose acetate film was shown to produce ^1^O_2_ under continuous exposure to room light for over 6 months [17]. In another study, a composite of polyvinyl chloride and porphyrins (namely: 5,10,15,20-tetrakis(4-methylphenyl)-21*H*,23*H*-porphine, 5,10,15,20-tetrakis(4-methoxyphenyl)-21*H*,23*H*-porphine, 5,10,15,20-tetra-4-pyridinyl-21*H*,23*H*-porphine, and 5,10,15,20-tetrakis(4-aminophenyl)-21*H*,23*H*-porphine) designed as a photo-disinfecting surface proved to be promising for effective and sustainable applications in water disinfection [110]. These examples illustrate the versatility and applicability of aPDT technology across different environments, showcasing its potential for long-term and sustainable microbial control in various settings.

Photodynamic self-disinfecting textiles, designed to possess intrinsic or integrated properties that enable them to effectively disinfect themselves, have also been reported. Efimov et al. developed phthalocyanine-impregnated cotton fabrics that were effective against viral, bacterial, and fungal pathogens and can be activated by moderate artificial, ambient, or natural light [111]. Textiles or fabric-based materials play a crucial role in various areas such as in healthcare settings, households, public facilities, agriculture, and industrial environments for containment, protection, and environmental management. For instance, nylon or polyester nets, provide containment for fish while allowing water exchange and waste removal [112,113]. In healthcare settings, textiles such as curtains, which create barriers between patient beds, and face masks play critical roles in controlling the spread of airborne pathogens. However, these textiles can also harbor various microorganisms, presenting transmission risks across different environments. Advancing this field, Li et al. developed self-antiviral fabrics demonstrating robust virucidal effects even under ultralow-power lamp light irradiation. These materials have shown remarkable washability and photostability, with the potential for over 100 uses without a loss in efficacy [114]. This durability and effectiveness indicate these textiles’ potential as a sustainable solution in different settings that demand rigorous hygiene standards [115]. Their use could significantly diminish the frequency of textile replacement, leading to lower operational costs and environmental impact.

However, the application of light-activated photosensitizers in creating self-disinfecting surfaces is still in its exploratory stages [116]. There remains a pressing need for comprehensive research into the feasibility, effectiveness, and safety of this technology. Understanding the environmental impacts, potential residues, and the overall sustainability of using such photodynamic materials is critical. Such investigations will not only help refine the technology but also ensure it can be safely and efficiently integrated into existing practices, ultimately enhancing infection control and sanitation in various sectors.

## 5. Advantages and Limitations of Photodynamic Self-Disinfecting Surfaces

The implementation of photodynamic self-disinfecting surfaces presents a promising approach to combat microbial infections and enhance overall health. However, like any technology, these surfaces come with their own set of advantages and limitations that must be carefully considered.

### 5.1. Advantages of Photodynamic Self-Disinfecting Surfaces

Photodynamic self-disinfecting surfaces have shown promise in healthcare facilities [117], dental treatments [118], public transport, and food processing facilities [119]. These offer numerous advantages since they are specifically tailored to the unique needs and challenges [120].

Photodynamic self-disinfecting surfaces have proven effective in pathogen control [121], attributed to generated ROS that are lethal to a wide range of pathogens, including bacteria, viruses, and fungi [12]. This capability is instrumental in preventing disease outbreaks and spread. Moreover, unlike intermittent chemical treatments, these surfaces offer continuous protection against microbial colonization and biofilm formation [35,121], which is vital for sustaining health.

In photodynamic self-disinfecting surfaces, PS are embedded within a polymeric material or encapsulated within a coating or thin film [104,122], minimizing their release into the environment. These surfaces control pathogens through a non-chemical process, thereby reducing reliance on harsh chemical disinfectants. This approach will not only reduce chemical residues in the water but will also lower the risk of cultivating microbial strains that are resistant to chemicals [5,123].

The design of self-disinfecting surfaces using aPDT can be made cost-effective by leveraging readily available and inexpensive light sources, such as LED lights or natural sunlight [13,85]. This approach, combined with the utilization of durable, efficient, eco-friendly, and economically viable PS, presents a highly sustainable disinfection solution [12]. For example, Zhang et al. created a novel nanofibrous membrane by blending polyacrylonitrile with the hydrophilic polymer poly (vinyl alcohol-co-ethylene) and integrating vitamin K. They fabricated this structure using the electrospinning technique, infusing it uniformly with vitamin K. The resulting membrane showcased impressive photoactivity, capable of generating ROS when exposed to both natural daylight and ultraviolet A (365 nm) light. This photoactive attribute allowed the membrane to rapidly achieve high levels of antimicrobial and antiviral effectiveness within less than 90 min [124]. The robustness of the membrane, attributed to electrospinning and its composite material formulation, ensures prolonged effectiveness. This durability implies infrequent replacement needs and, therefore, reduced long-term operational costs. Furthermore, to enhance cost-efficiency, PSs could be encapsulated within coatings that enhance their adherence to diverse surfaces or embedded directly into commonly used materials in construction and manufacturing, such as plastics, ceramics, and textiles [125,126,127]. This integration would increase the longevity and wear resistance of the surfaces.

The capability for continuous disinfection reduces the frequency and intensity of manual cleaning and maintenance, thereby saving on labor and materials [128]. Continuous disinfection can prevent biofouling and microbial corrosion, significantly increasing the durability of equipment and infrastructure, and minimizing costs related to frequent replacements and repairs [129]. Thus, leveraging these technologies can substantially enhance economic efficiency and sustainability in maintaining sterile environments.

By harnessing the power of light and advancements in material science, photodynamic self-disinfecting surfaces can offer a cutting-edge solution to some of the most pressing microbial challenges.

### 5.2. Limitations of Photodynamic Self-Disinfecting Surfaces

Photodynamic self-disinfecting surfaces offer significant potential for reducing microbial contamination, but they also face notable challenges. A thorough understanding of these challenges is essential for refining the technology and overcoming inherent drawbacks.

One major factor influencing the efficacy of these surfaces is their dependence on sufficient light exposure to activate the photosensitizers [13]. In environments where light may be patchy or blocked, such as in shaded areas or beneath water surfaces, the antimicrobial effectiveness of these surfaces can be markedly diminished [12]. However, innovative approaches can be developed to address this challenge.

Photodynamic self-disinfecting surfaces are designed to target infectious microorganisms. However, their lack of selectivity can unintentionally damage unintended targets [20]. Therefore, optimizing the selectivity of photodynamic self-disinfecting surfaces to minimize collateral damage remains a significant challenge in their application.

Furthermore, the effectiveness of photodynamic surfaces can vary against different microbes. Some pathogens may be susceptible to the ROS these surfaces produce, while others may show resistance, potentially leading to incomplete disinfection [130]. Therefore, optimizing the performance of photodynamic surfaces requires a comprehensive understanding of the diverse microbial landscape they may encounter, as well as strategies to address potential resistance mechanisms.

Addressing these limitations comprehensively is crucial to enhancing the practicality and widespread adoption of photodynamic self-disinfecting surfaces in various sectors.

## 6. Conclusions and Future Perspectives

Antimicrobial photodynamic therapy using self-disinfecting surfaces represents a promising approach to combat microbial infections across various settings. These surfaces utilize a diverse range of PSs and light sources to effectively target bacteria, viruses, and fungi by activating embedded PSs within polymers or coatings using light energy.

The ongoing advancement of aPDT self-disinfecting surfaces holds significant potential driven by improvements in PSs and light source technologies. These innovations can enhance specificity and efficacy against specific pathogens while mitigating potential environmental and human health risks. Tailored approaches that optimize PSs and light wavelengths can broaden applications in healthcare facilities, public spaces, and transportation systems where microbial contamination poses significant challenges.

Incorporating aPDT into routine infection control measures offers the promise of safer environments by reducing the transmission of infectious agents. Future research should prioritize optimizing surface treatments, enhancing the scalability of production, and ensuring long-term durability to facilitate practical implementation and sustainability of these advanced disinfection technologies.

Effective and sustainable implementation of photodynamic self-disinfecting surfaces requires collaborative efforts across disciplines to establish strong standards, policies, and regulatory frameworks. These efforts are crucial to support the global adoption and integration of aPDT technologies in infection control strategies.

## Figures and Tables

**Table 1:** Microorganisms, PSs, light sources, and polymers/coatings in aPDT self-disinfecting surfaces.

Microorganism	PSs Used	Light Source	Self-Disinfecting Surface	Polymer/Coating	Ref.
Bacteria					
*Pseudomonas aeruginosa*	Rose Bengal	White light LED lamps (400–700 nm, 72 J/cm^2^)	Thin films	Poly (2-hydroxyethyl methacrylate)	[29]
		Green light of 515 nm; dose was 120 J/cm^2^	Ion exchange resins	Amberlite^®^ IRA-900 or Amberlite^®^ IRA-400	[30]
		Light-emitting diode lamp (LED) (515 ± 10 nm, 200 J/cm^2^)	Ion exchange resins	Amberlite^®^ IRA-900 or Amberlite^®^ IRA-400	[31]
	Rose Bengal and tetrabutylammonium iodide	white light irradiation (400–700 nm, 24 J/cm^2^)	Thin film surface	Poly (2-hydroxyethyl methacrylate)	[32]
	Graphitic carbon nitride (metal-free)	Visible LED light (417 nm, 160 W /m^2^)	Cotton fabrics	Graphitic carbon nitride	[33]
*Staphylococcus aureus*	Zinc (II) 5,10,15,20-tetrakis((*N*-4-[3-(trifluoromethyl)-3*H*-diazirin-3-yl] benzyl)-4-pyridyl)-21*H*,23*H*-porphine tetrabromide	white LED light: dose was 59.37 J/cm^2^	Polyethylene terephthalate (PET) coupon surface	Polyethylene terephthalate	[34]
	Zinc (II) 5,10,15,20-tetrakis(*N*-methyl-4-pyridyl)-21*H*,23*H*-porphine tetraiodide				
	Zinc-tetra(4-*N*-methylpyridyl) porphine (ZnTMPyP^4+^)	Visible light (400–700 nm, 65 ± 5 mW/cm^2^)	Vescom materials	*N*-methyl-4(4′-formyl-styryl) pyridinium methosulfate acetal poly (vinyl alcohol)	[35]
	Rose Bengal				
	Methylene blue				
	Methylene Blue	Laser light (100 mW, 660 nm, 42.1 J/cm^2^)	Dextran-graft-polyacrylamide hydrogels material	Dextran-graft-polyacrylamide	[36]
	Zinc and copper phthalocyanines (ZnPc and CuPc)	Light (7.2 J/cm^2^)	Glass surfaces	3,4-ethylenedioxythiophene and poly (3,4-ethylenedioxythiophene	[37]
	Thionine	Xenon lamp (500 W, 400–700 nm, 65 ± 5 mW/cm^2^	PET/cotton blended fabrics	Polyethylene terephthalate	[38]
	Toluidine blue O	Artificial sun irradiation (250 W/m^2^)	Packaging material surface	Tempo-oxidized carbon nanofibers	[39]
		LED light (450–700 nm, 6.57 mW/cm^2^, and 94.6 mJ/cm^2^)	Polymer surface	Polyether block amide	[40]
	Rose Bengal	Light-emitting diode lamp (LED) (515 ± 10 nm, 200 J/cm^2^	Ion exchange resins	Amberlite^®^ IRA-900 or Amberlite^®^ IRA-400	[31]
		Visible light (Xenon lamp) (420 nm, 35 mW/cm^2^)	Cotton fabric	Europium and dysprosium co-doped SrAl_2_O_4_ phosphor on nylon and *N*-methyl-4(4′-formylstyryl) pyridinium methosulfate acetal poly (vinyl alcohol)	[15]
		Red LED (480 nm, 200 mW, and 526 mW/cm^2^)	Denture base material	Polymethyl methacrylate	[41]
	BODIPY (2,6-diiodo-1,3,5,7-tetramethyl-8-(2,6-dichlorophenyl)-4,4′-difluoroboradiazaindacene)	Light (520 nm, 2.4 mW/cm^2^, 52.1 J/cm^2^	Polylactide surface coating	Monomethoxy poly (ethylene glycol)-poly (lactic acid)	[42]
	Chlorophyllin (E140ii)	Visible light (400–700 nm, 80 ± 5 mW/cm^2^	Cotton fabric	Polyacrylonitrile	[28]
*S. epidermidis*	Toluidine blue O	LED light (450–700 nm, 6.57 mW/cm^2^, and 94.6 mJ/cm^2^)	Polymer surface	Polyether block amide	[40]
*Streptococcus pneumoniae*	Tetra(4-carboxyphenyl) porphyrin linked to hexafluorophosphate (TTCP-PF6)	Ultra-low power (20 W/m^2^) white light or simulated sunlight irradiation	Face masks	-	[43]
*E. coli*	9-Aminoacridine	Illuminated for 19 h using 200 W spotlight (wavelength from 380 nm to 750 nm)	Stainless steel surface	Poly (*N*-methacryloyl 3,4-dihydroxy-L-phenylalanine methyl ester)-b-poly (2-methacryloxyethyltrimethylammonium chloride) copolymer	[44]
	Azure A (AA) and 5-(4-aminophenyl)-10,15,20-(triphenyl)porphyrin (APTPP)	Xenon lamp or diode laser (445 for APTPP and 638 nm for AA)	Glass Surface	-	[45]
	Zinc and copper phthalocyanines (ZnPc and CuPc)	Light (7.2 J/cm^2^)	Glass surfaces	3,4-ethylenedioxythiophene and poly (3,4-ethylenedioxythiophene	[37]
	Graphitic carbon nitride (metal-free)	Visible LED light (417 nm, f 160 W/m^2^)	Cotton fabrics	Graphitic carbon nitride	[33]
	Thionine	Xenon lamp (500 W, 400–700 nm, 65 ± 5 mW/cm^2^	PET/cotton blended fabrics	Polyethylene terephthalate	[38]
	Toluidine blue O	Artificial sun irradiation (250 W/m^2^)	Packaging material surface	Tempo-oxidized carbon nanofibers	[39]
		LED light (450–700 nm, 6.57 mW/cm^2^, and 94.6 mJ/cm^2^)	Polymer surface	Polyether block amide	[40]
	Rose Bengal	Light-emitting diode lamp (LED) (515 ± 10 nm, 200 J/cm^2^	Ion exchange resins	Amberlite^®^ IRA-900 or Amberlite^®^ IRA-400	[31]
	Rose Bengal and tetrabutylammonium iodide	white light irradiation (400–700 nm, 24 J/cm^2^)	Thin film surface	Poly (2-hydroxyethyl methacrylate)	[32]
*Enterococcus faecalis*	Rose Bengal	Light-emitting diode lamp (LED) (515 ± 10 nm, 200 J/cm^2^	Ion exchange resins	Amberlite^®^ IRA-900 or Amberlite^®^ IRA-400	[31]
*Acinetobacter baumannii*	Toluidine blue O	LED light (450–700 nm, 6.57 mW/cm^2^, and 94.6 mJ/cm^2^)	Polymer surface	Polyether block amide	[40]
Viruses					
Human coronavirus strain HCoV-229E	Zinc-tetra(4-*N*-methylpyridyl) porphine	Visible light (400–700 nm, 65 ± 5 mW/cm^2^)	Vescom materials	*N*-methyl-4(4′-formyl-styryl) pyridinium methosulfate acetal poly (vinyl alcohol).	[35]
	Methylene blue				
	Rose Bengal				
	Thionine	LumaCare PDT light (400–700 nm, 65 ± 5 mW/cm^2^)	PET/cotton-blended fabrics	Polyethylene terephthalate	[38]
*Bacteriophage Qβ*	Phenoxy-substituted zinc phthalocyanine	Visible light (room light)	Films	Cellulose acetate coating active	[17]
*Influenza A virus H1N1 strain*	Tetra(4-carboxyphenyl) porphyrin linked to hexafluorophosphate (TTCP-PF6)	Ultra-low power (20 W/m^2^) white light or simulated sunlight irradiation	Face masks	-	[43]
Fungi					
*Candida albicans*	Rose Bengal	Light-emitting diode lamp (LED) (515 ± 10 nm, 200 J/cm^2^	Ion exchange resins	Amberlite^®^ IRA-900 or Amberlite^®^ IRA-400	[31]
		Red LED (480 nm, 200 mW, and 526 mW/cm^2^)	Denture base material	Polymethyl methacrylate	[41]
